# Retrieving the intravascular ultrasound catheter in the coronary artery by a gooseneck snare: a case report

**DOI:** 10.1093/ehjcr/ytaf530

**Published:** 2025-10-14

**Authors:** Hiroshi Fujita, Ken Ito, Kazuomi Ono, Nobuyuki Ohte

**Affiliations:** Department of Cardiology, Gamagori City Hospital, 1-1 Mukoda Hirata-cho, Gamagori 443-8501, Japan; Department of Cardiology, Gamagori City Hospital, 1-1 Mukoda Hirata-cho, Gamagori 443-8501, Japan; Department of Cardiology, Gamagori City Hospital, 1-1 Mukoda Hirata-cho, Gamagori 443-8501, Japan; Department of Cardiology, Gamagori City Hospital, 1-1 Mukoda Hirata-cho, Gamagori 443-8501, Japan; Department of Cardiology, Toyokawa City Hospital, Toyokawa City, Aichi 442-8561, Japan

**Keywords:** Gooseneck snare, Entrapment of devices, Coronary artery

## Abstract

**Background:**

Device rupture during percutaneous coronary intervention (PCI) is a rare complication. However, devices remaining in the coronary artery must be removed promptly because they can cause occlusion. We report our success in retrieving a fractured intravascular ultrasound (IVUS) catheter using a gooseneck snare.

**Case summary:**

A 78-year-old man presented with repeated chest pain on effort. Coronary angiography showed a significant stenosis in the peripheral left circumflex artery (LCX). We performed PCI for this lesion. After pre-dilating the lesion with a balloon, we tried to observe the coronary arterial lumen with an IVUS catheter. At that time, the IVUS catheter was ruptured, and the distal imaging core shaft of it remained in the LCX. We used a gooseneck snare to retrieve it; however, we were unable to advance it to the target site using the conventional method for delivery because of the tortuous LCX. Therefore, we attempted our modified method, using the snare catheter as a microcatheter with a guidewire, to insert the snare wire loop into the target site and successfully retrieved the remaining distal imaging core shaft. After the procedure, we confirmed that the LCX was not visually injured.

**Conclusion:**

Using a snare catheter, such as a microcatheter, is a beneficial option for delivering a gooseneck snare to the target in the coronary artery.

Learning points

- Rupture of the intravascular ultrasound catheter in coronary arteries is rare but has been encountered.
- Using a gooseneck snare catheter as a microcatheter for retrieving devices in the coronary artery is a novel strategy.

## Introduction

Rupture or entrapment of devices, such as a guidewire, a balloon catheter, or an intravascular ultrasound (IVUS) catheter, during percutaneous coronary intervention (PCI) is rare, with a reported incidence of 0.1%–0.8%.^1,2^ However, it could be fatal due to coronary artery occlusion. Therefore, we must retrieve the devices immediately after the accident, possibly using percutaneous techniques.^3–6^ If this attempt fails, the patient will require highly invasive surgical treatment.^7,8^ Emergent management of this complication is mandatory, and percutaneous techniques are the first line for bailout. We report a case of a fractured IVUS catheter during PCI that was successfully retrieved using a modified gooseneck snare method.

## Summary figure

**Figure:**
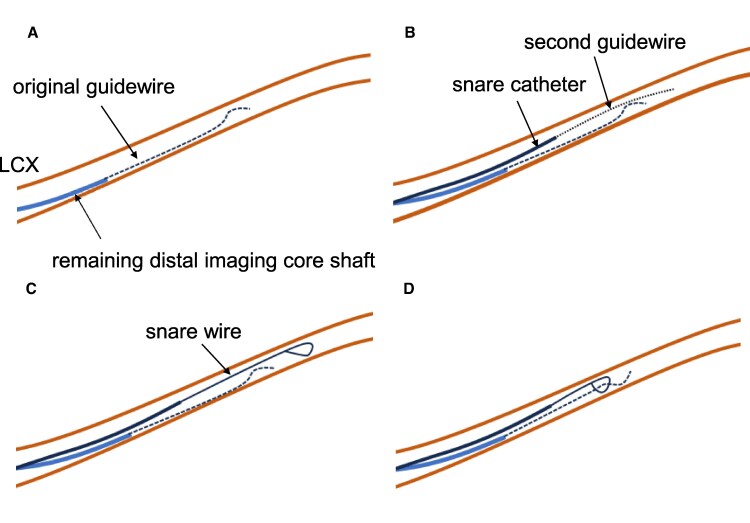


## Case presentation

**Figure 1:**
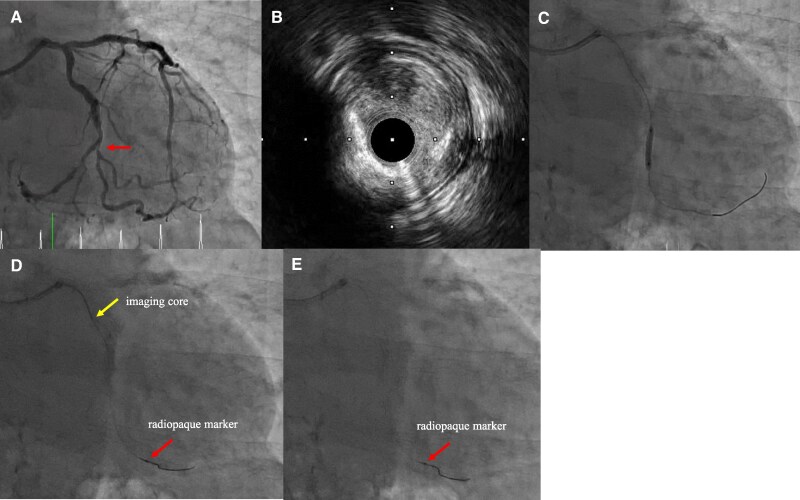
Coronary angiography and intravascular ultrasound findings. (*A*) The target lesion is shown with a arrow. (*B*) The intravascular ultrasound finding of the target lesion shows fibrous plaque with mild calcification. (*C*) Pre-dilatation of the target lesion using a balloon. (*D*) The intravascular ultrasound imaging core (arrow) is located abnormally distant from the radiopaque marker (arrow). (*E*) The radiopaque maker remains in the coronary artery after pulling out the main body of the intravascular ultrasound catheter.

**Figure 2:**
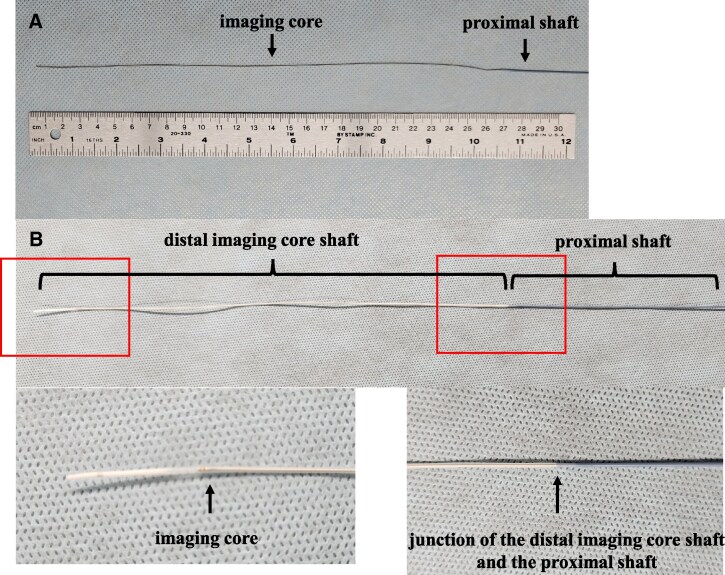
The structure of the intravascular ultrasound catheter. (*A*) The top part of the broken intravascular ultrasound catheter. With a distal imaging core shaft loss, the imaging core is exposed to the outside of the shaft. (*B*) Upper: an appearance of a normal intravascular ultrasound catheter. Lower left: the tip of the imaging core in the distal imaging core shaft. Lower right: the junction of the distal and proximal core shafts.

**Figure 3:**
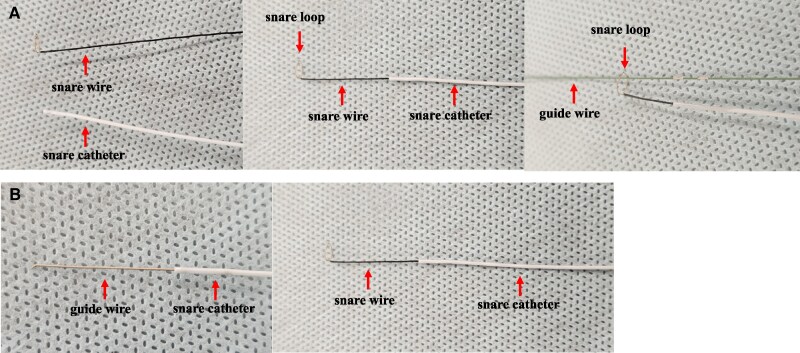
Appearances and how to use a gooseneck snare. (*A*) Left: a snare wire and a snare catheter. Center: a whole appearance of a gooseneck snare. A snare wire is taken out from the tip of the snare catheter. Right: the usual way to use the snare wire and snare catheter along the guidewire. The guidewire goes through the snare wire loop, and the snare catheter and the guidewire are not coaxial. Initially, crossing the stenosis lesion was attempted using this system, but it failed. (*B*) The second guidewire was inserted into the snare catheter instead of a snare wire. The snare catheter is used as a microcatheter. The snare catheter and the guidewire are coaxial. The microcatheter crossed the stenotic lesion successfully and advanced beyond the remaining distal imaging core shaft. Then, the guidewire was exchanged for a snare wire.

**Figure 4:**
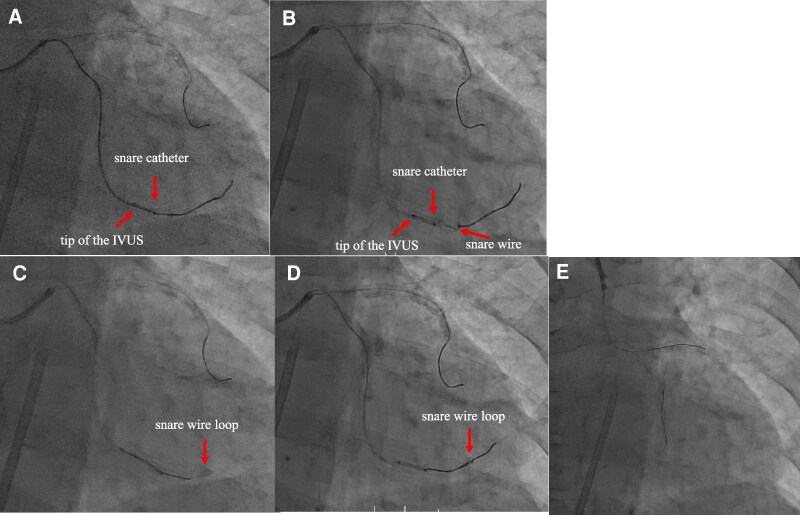
Retrieval of the remaining intravascular ultrasound distal imaging core shaft. (*A* and *B*) Delivery of the snare catheter to the target site using the modified method. (*C*) Manipulate the original guidewire loading into the distal imaging core shaft to pass the gooseneck snare wire loop. (*D*) The original guidewire goes through the loop. (*E*) After retrieval of the intravascular ultrasound distal imaging core shaft.

**Figure 5:**
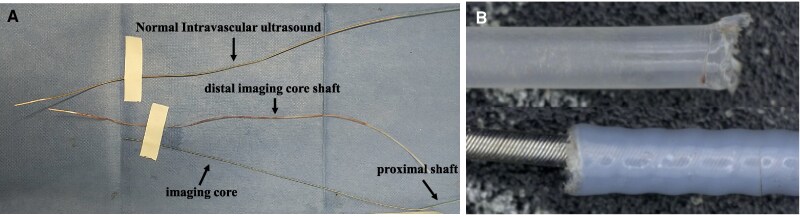
The broken intravascular ultrasound catheter. (*A*) The picture shows the normal (upper) and fractured (lower) intravascular ultrasound shafts. (*B*) A magnified picture of the broken site.

A 78-year-old male visited the hospital with exertional chest pain. Because his symptoms frequently occurred with light exertion, we performed coronary angiography. It showed a severe stenosis lesion in the middle portion of the left circumflex artery (LCX) (*Figure 1A*). We performed a fractional flow reserve examination, and the result was positive for myocardial ischaemia at 0.74. Thus, we decided to perform PCI. We chose a 7 Fr Hyperion SPB guiding catheter (ASAHI-Intech, Seto, Japan) and inserted a Route 0.014-inch guidewire (ASAHI-Intech) into the LCX. First, we performed an IVUS examination (OrtiCross, Boston Scientific, Massachusetts, USA) and found a fibrous plaque with mild calcification (*Figure 1B*). We predilated the lesion with a 2.5 × 13 mm Aperta NSE (NIPRO, Osaka, Japan) (*Figure 1C*). When we performed a second IVUS to determine the stenting site, the fluoroscopy revealed that the IVUS imaging core was positioned distant from the radiopaque marker on the distal imaging core shaft (*Figure 1D*). We attempted to rotate the IVUS imaging core by operating the motor drive unit, but were unsuccessful in doing so. When we pulled the IVUS catheter, the fluoroscopy showed no movement of the radiopaque marker on the distal imaging core shaft; however, the main body of the IVUS catheter was removed from the guide catheter (*Figure 1E*). We found no distal imaging core shaft (*Figure 2A*); the imaging core should be in it (*Figure 2B*). The IVUS imaging core shaft was suspected to be broken at the junction between the distal and the proximal core shafts. The distal imaging core shaft was suspected to remain in the LCX. We attempted to remove it using a 4 mm gooseneck snare (Goose Neck Microsnare: Medtronic, Minnesota, USA). A gooseneck snare consists of a snare wire and a snare catheter, with the snare wire stored within the snare catheter. In the standard method, when delivering a gooseneck snare, we inserted the second guidewire into the LCX along the original guidewire, which was loaded into the distal imaging core shaft. Then, we passed the snare wire loop taken out from the tip of the snare catheter through the second guidewire at hand and tried to advance the gooseneck snare along the second guidewire in the guiding catheter (*Figure 3A*). However, we could not proceed with the gooseneck snare because of the tortuous LCX. So, we attempted our modified method. We inserted the second guidewire into the snare catheter and used it like a microcatheter (*Figure 3B*). Using this modified method, we successfully advanced the snare catheter beyond the tip of the remaining IVUS distal imaging core shaft (*Figure 4A*). Then, we exchanged the guidewire with the snare wire (*Figure 4B*). Successively, we manipulated the original guidewire loading into the distal imaging core shaft to pass the snare wire loop beyond the target (*Figure 4C*) and succeeded in inserting the original guidewire into the snare wire loop (*Figure 4D*). Then, we grabbed the ruptured IVUS distal imaging core shaft by the snare wire loop while pulling back the snare loop along the original guidewire, and we successfully removed it from the LCX (*Figure 4E*). The fractured distal imaging core shaft is shown in *Figure 5*. After retrieving it, we confirmed that the LCX was not visually injured. The vendor’s inspection was unable to identify the cause of the material fracture.

## Discussion

Coronary interventionists should remember that device-related troubles in the coronary artery can occur over time^1–8^ and be proficient in the bailout method. In this report, we describe a case of IVUS catheter rupture in which the catheter remained in the coronary artery, and we successfully retrieved it using our modified gooseneck snare method. To our knowledge, this is the first report of a new method. In the present case, we were unable to advance the gooseneck snare to the target site using the standard method due to the tortuosity of the LCX. Therefore, we attempted to use a snare catheter as a microcatheter, guided by a guidewire, to reach the target site. In the standard method, a second guidewire is passed through the snare loop at hand, which often cannot be delivered to the target site in small-diameter vessels such as coronary arteries. In our modified procedure, the snare catheter is used like a microcatheter, allowing for the smooth delivery of the catheter to the target site, even in cases where the coronary artery exhibits tortuosity and calcification.

## Conclusion

Using a snare catheter, such as a microcatheter with a guidewire, is a beneficial option for delivering a gooseneck snare to the target in the coronary arteries.

## Lead author biography


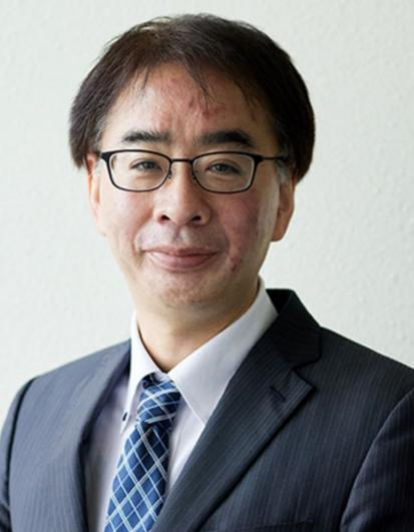


Hiroshi Fujita is an interventional cardiologist and the Second Chief of the Department of Cardiology at Gamagori City Hospital. He had interventional training at Toyohashi Heart Center, Toyohashi, Japan, from 2005 to 2009.

## Data Availability

All data are available upon request.

## Notes

**Consent:** The authors confirm that written consent for the submission and publication of this case report, including images and associated text, has been obtained from the patient, in line with the COPE guidelines.

**Funding:** None declared.
